# Quality of Hospital Electronic Health Record (EHR) Data Based on the International Consortium for Health Outcomes Measurement (ICHOM) in Heart Failure: Pilot Data Quality Assessment Study

**DOI:** 10.2196/27842

**Published:** 2021-08-04

**Authors:** Hannelore Aerts, Dipak Kalra, Carlos Sáez, Juan Manuel Ramírez-Anguita, Miguel-Angel Mayer, Juan M Garcia-Gomez, Marta Durà-Hernández, Geert Thienpont, Pascal Coorevits

**Affiliations:** 1 Medical Informatics and Statistics Unit, Department of Public Health and Primary Care Faculty of Medicine and Health Sciences Ghent University Ghent Belgium; 2 The European Institute for Innovation through Health Data (i~HD) Ghent Belgium; 3 Biomedical Data Science Lab Instituto Universitario de Tecnologías de la Información y Comunicaciones Universitat Politècnica de València Valencia Spain; 4 Research Programme on Biomedical Informatics Hospital del Mar Medical Research Institute and Universitat Pompeu Fabra Barcelona Spain; 5 Research in Advanced Medical Informatics and Telematics (RAMIT) Ghent Belgium

**Keywords:** data quality, electronic health records, heart failure, value-based health insurance, patient outcome assessment

## Abstract

**Background:**

There is increasing recognition that health care providers need to focus attention, and be judged against, the impact they have on the health outcomes experienced by patients. The measurement of health outcomes as a routine part of clinical documentation is probably the only scalable way of collecting outcomes evidence, since secondary data collection is expensive and error-prone. However, there is uncertainty about whether routinely collected clinical data within electronic health record (EHR) systems includes the data most relevant to measuring and comparing outcomes and if those items are collected to a good enough data quality to be relied upon for outcomes assessment, since several studies have pointed out significant issues regarding EHR data availability and quality.

**Objective:**

In this paper, we first describe a practical approach to data quality assessment of health outcomes, based on a literature review of existing frameworks for quality assessment of health data and multistakeholder consultation. Adopting this approach, we performed a pilot study on a subset of 21 International Consortium for Health Outcomes Measurement (ICHOM) outcomes data items from patients with congestive heart failure.

**Methods:**

All available registries compatible with the diagnosis of heart failure within an EHR data repository of a general hospital (142,345 visits and 12,503 patients) were extracted and mapped to the ICHOM format. We focused our pilot assessment on 5 commonly used data quality dimensions: completeness, correctness, consistency, uniqueness, and temporal stability.

**Results:**

We found high scores (>95%) for the consistency, completeness, and uniqueness dimensions. Temporal stability analyses showed some changes over time in the reported use of medication to treat heart failure, as well as in the recording of past medical conditions. Finally, the investigation of data correctness suggested several issues concerning the characterization of missing data values. Many of these issues appear to be introduced while mapping the IMASIS-2 relational database contents to the ICHOM format, as the latter requires a level of detail that is not explicitly available in the coded data of an EHR.

**Conclusions:**

Overall, results of this pilot study revealed good data quality for the subset of heart failure outcomes collected at the Hospital del Mar. Nevertheless, some important data errors were identified that were caused by fundamentally different data collection practices in routine clinical care versus research, for which the ICHOM standard set was originally developed. To truly examine to what extent hospitals today are able to routinely collect the evidence of their success in achieving good health outcomes, future research would benefit from performing more extensive data quality assessments, including all data items from the ICHOM standards set and across multiple hospitals.

## Introduction

Increasing quantities of health data are being collected across care organizations, creating a powerful opportunity to learn from these data how to improve patient care and accelerate research. The earliest call to action and formalized approach for using health data to assess quality of care was probably the Donabedian model of quality [1]. He categorized the assessment of health care quality under structure (how services are organized and resourced), process (how care is delivered and what care activities are undertaken), and outcome (what health impact it has). Over the decades, it has proved much easier to develop and implement audits of structure or process, but formalized assessments of outcome appear to be more challenging because it is harder to define what we mean by outcomes and how best to measure them [2]. A formalized approach to measuring health outcomes was proposed by Porter and Teisberg [3], within their model of the assessment of “value” in a seminal publication in 2006. Within this value equation, outcomes were defined as “the outcomes that matter to patients and the costs to achieve those outcomes” [3]. This “Value-Based Health Care” model has grown into a portfolio of health outcomes standards for measuring value, developed and promoted by the International Consortium for Health Outcomes Measurement (ICHOM). These health outcomes standards, formalized as indicators to be collected, quantified, and compared between health care providers, have stimulated a global interest in benchmarking and comparing health outcomes [4].

All these models hinge upon the essential ability to measure health, health care, and its outcomes. Health data are therefore a vital ingredient. To enable accurate measurement, data have to be captured and represented to a high quality. Unreliable data, such as incomplete, incorrect, or missing data entries, will inevitably lead to biased analyses, resulting in misdirected efforts to improve quality or false research interpretations.

Yet, several studies have pointed out significant issues regarding availability and quality of electronic health record (EHR) data [5-10]. For example, the “Electronic Health Records for Clinical Research” project, funded by the Innovative Medicine Initiative, clearly demonstrated that many variables, among which even fundamental ones such as patient weight, are frequently not present within EHR systems [8]. Incorrect or absent recording of patient weights, though, can lead to medication dosage errors. Hirata and colleagues [11] examined the frequency and consequences of weight errors that occurred across 79,000 emergency department encounters of children under the age of 5 years. They revealed that, although weight errors were relatively rare (0.63%), a large proportion of weight errors led to subsequent medication-dosing errors (34%). An earlier study by Selbst and colleagues [12] also investigated the consequences of medication errors in a paediatric emergency department. They found that almost half of patients required additional monitoring (30%), examination (6%), or treatment (12%) after medication errors resulting from weight errors. To obtain reliable outcome measures from routinely collected EHR data, Sáez et al [10] developed a national, standardized, data quality–assessed, integrated data repository on maternal-child care. During this process, they found that variability in data quality across hospital sites could lead to imprecise comparison of measurements. Moreover, data quality indices, the efficiency of research processes, and the reliability of subsequent results have been found to improve if patient records are assessed for data quality [13,14]. Hence, quality assessment of source health data is crucial to identify and mitigate data quality problems for proper data use and reuse.

In this paper, we first describe our practical approach to quality assessment of health outcomes data. Adopting this methodology, we performed a pilot study on a subset of ICHOM outcomes data collected during routine clinical care of patients with congestive heart failure (CHF) in a general hospital, given the high prevalence and margin for outcomes improvement in heart failure [15]. Assessing data quality of outcomes data obtained during routine clinical care is of great interest since ICHOM indicators are currently collected through dedicated data collection into specialist outcome measurement systems, which results in useful data but is not a scalable process. The complexity of the analysis and in selecting the diagnosis for more than one condition, as well as the comorbidities associated with each disease, the different treatments received in each case, and all the variables used in the analysis, make it very difficult to conduct a system-wide quality assessment including several diseases and to interpret the results of a multiple disease analysis.

## Methods

### Data Quality Assessment

Research into data quality has gained attention since the seminal work by Wang and Strong [16], who proposed a comprehensive “fit-for-use” data quality assessment framework using data quality dimensions. Since then, several studies have aimed to define data quality dimensions and methodologies to describe and measure the complex multidimensional aspects of data quality [14,17-20]. Across studies, little agreement exists about the exact definition and meaning of data quality dimensions. Despite differences in terminology, though, many of the proposed dimensions and solutions aim to address conceptually similar data quality features [14]. 

Following a review of existing literature, the data quality task force of the European Institute for Innovation through Health Data (i~HD) [21] identified 9 frameworks for quality assessment of health data [5,14,19,22-27]. From these frameworks, 9 data quality dimensions were selected during a series of workshops with clinical care, clinical research, and information and communication technology leads from 70 European hospitals: completeness, consistency, correctness, uniqueness, stability, timeliness, trustworthiness, contextualization, and representativeness. The selected data quality dimensions were deemed most important to assess the quality of health data if these data are to be useful for patient care, organizational learning (quality improvement, such as the assessment of health outcomes), and research (big data research and case finding for clinical trial recruitment). Multimedia Appendix 1 provides an overview of the selected data quality dimensions, together with their original terminology; the completeness, consistency, correctness, uniqueness, and stability dimensions were the most commonly used in the data quality literature, and for this reason, we selected them for the quality assessment in this study [14,20]. For instance, trustworthiness and timeliness are based on some types of metadata that are not usually available or accessible in EHR. Although sometimes the first 3 can overlap in their definitions or be contained within each other, we prefer making them orthogonal. For instance, a patient observation is incomplete if it is not registered, inconsistent if it does not comply with formatting requirements, or incorrect if it is unlikely to be true for a specific patient. For example, multiple normal kidney blood test results for a patient on dialysis would be consistent, though incorrect. Uniqueness, in turn, assesses whether duplications are present among patient records, for example as a result of an incomplete merging of patient records between hospital departments.

Further, stability relates to the probabilistic concordance of data among different data sources such as hospitals, physicians, or devices or over time [28]. For example, variability among centers has been found in liver offer acceptance rates for pediatric patients and cannot be explained by donor and recipient factors [29]. In some cases, standardization of procedures and analyses can reduce levels of variability. However, sometimes differences among centers persist even when using standard procedures, for instance, between diffusion tensor magnetic resonance imaging findings obtained at different acquisition centers using a standard protocol [30]. Likewise, when data are collected over time, temporal changes can occur due to several reasons, including changes in clinical practice or coding scheme used in the EHR [31].

Next, timeliness describes how promptly information is processed or how current recorded information is, for instance, to evaluate whether a current medication list within an EHR system is up to date or if there is a delay in updating this from a pharmacy subsystem. Trustworthiness relates to the availability of registry governance metadata and the data owner’s reputation. For example, it must be possible for someone accessing a health data item or clinical document to confidently know when and where it was captured, by whom, and if it has been modified since the original entry. Further, contextualization relates to whether the data are annotated with their acquisition context, which can be crucial for correct interpretation of the results, for instance, whether blood glucose laboratory results were obtained while the patient was fasting. Finally, representativeness captures whether a dataset is representative for the population from which it is supposed to be drawn, in order to allow valid inference. 

### Pilot Assessment

#### Dataset 

For this pilot assessment, we used data from the Parc Salut Mar Barcelona, a complete health care services organization with its information system database (IMASIS) as EHR. IMASIS includes and shares clinical information from 2 general hospitals, 1 mental health care center, 1 social health care center, and 5 emergency rooms in the Barcelona city area (Spain). IMASIS contains clinical information from approximately 1.5 million patients who have used the services of this health care system since 1989, across different settings such as admissions, outpatient consultations, emergency room visits, and major ambulatory surgery appointments. IMASIS-2 is the anonymized relational database of IMASIS that was created during the European Medical Information Framework (EMIF) project [32] and is the data source used for research purposes. It contains structured data related to diagnosis, procedures, drug administration, and laboratory tests and clinical annotations in a free-text format. Since natural language processing falls beyond the scope of this project, we only used structured data. The study protocol was approved by the Ethics Committee of Parc Salut Mar (num. 2016/6935/I), under the research activities related to ischemic heart disease carried out during the EMIF project funded by the Innovative Medicines Initiative.

As a case study, data from patients diagnosed with CHF were used. Heart failure is a chronic condition, severely impacting people’s quality of life. With a prevalence of over 23 million worldwide, it poses a significant public health problem [33]. Collecting meaningful data on the health status of heart failure patients is therefore an important step to ensure better quality care and as a result, better quality of life for these patients.

**Figure 1:**
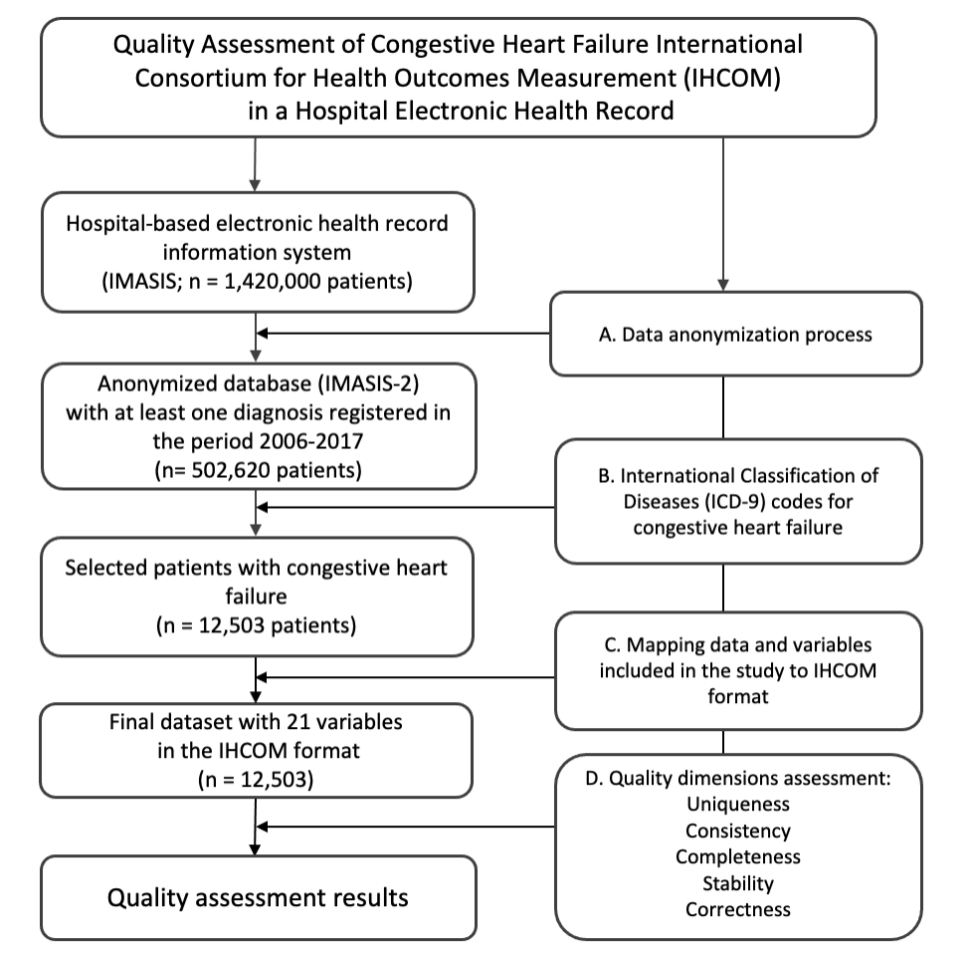
Overview of the procedure to identify the patients to generate the study dataset.

All patients (n=502,620) who attended the hospital at least once between January 1, 2006 and November 7, 2017 and who had at least one diagnosis entry of CHF were extracted from the IMASIS-2 database. Specifically, the selection of patients was based on the following diagnosis codes of the International Classification of Diseases ninth edition (ICD-9): 428, 428.0, 428.1, 428.2, 428.20, 428.21, 428.22, 428.23, 428.3, 428.30, 428.31, 428.32, 428.33, 428.4, 428.40, 428.41, 428.42, 428.43, 428.9. In total, the dataset included 142,345 patient visit records describing the medical history of 12,503 different patients who had one or more of these diagnoses. Figure 1 provides a flow diagram of the different steps that were performed to obtain our study dataset. The main steps followed in the study were (1) a data anonymization process, (2) selection of the ICD-9 codes to select patients with CHF, (3) mapping data and variables included in the study to the IHCOM standard format, and (4) quality dimensions analysis.

The ICHOM heart failure outcomes standard set [13] was chosen as the most appropriate source of outcome indicators to target. Of the total of 72 ICHOM data items, a subset of 21 variables was selected as being most likely to be routinely collected within the hospital for patients with CHF and to be indicative of the overall quality of data collected for this type of patient. In addition, these variables allowed us to have complete information for the main characteristics of patients including age and sex as well as relevant comorbidities, such as hypertension or diabetes mellitus, and some of the most frequent treatments received for CHF, such as beta blockers, diuretics, and digoxin. The 21 variables were organized in 6 areas: identifiers, demographic factors, baseline health status, treatment variables, burden of care, and mortality. In addition, a visit identifier was included to distinguish different patient visit records. An overview of all variables included in the pilot assessment can be found in Table 1. In addition, Multimedia Appendix 2 shows the ICD-9 codes used to identify baseline health status variables, and Multimedia Appendix 3 shows the Anatomical Therapeutic Chemical classification system codes of the World Health Organization [34] to retrieve patients’ medication usage. 

**Table 1:** Overview of International Consortium for Health Outcomes Measurement (ICHOM) variables used in the pilot assessment.

Item			Definition		Response options
**Identifiers**					
	Patient ID	Patient’s medical record number		According to institution	
	Visit ID	Unique visit record identifier		Not included in the ICHOM standard set	
**Demographic factors**					
	Age	Date of birth		DD/MM/YYYY	
	Sex	Sex at birth		1=Male, 2=Female	
**Baseline health status**					
	Atrial fibrillation	Ever diagnosed with atrial fibrillation		0=No, 1=Yes, 999=Unknown	
	Prior myocardial infarction	Ever diagnosed with myocardial infarction		0=No, 1=Yes, 999=Unknown	
	Hypertension	History of hypertension		0=No, 1=Yes, 999=Unknown	
	Diabetes mellitus	Ever diagnosed with diabetes mellitus		0=No, 1=Yes, 999=Unknown	
	Echocardiogram performed	Echocardiogram performed to assess ejection fraction		0=No, 1=Yes, 999=Unknown	
	Height	Height (cm)		Numeric value of height in the metric system	
	Weight	Weight (kg)		Numeric value of weight in the metric system	
	Alcohol use	Consumption of >1 alcoholic drink a day		0=No, 1=Yes, 999=Unknown	
	Smoking status	Current smoking status		0=No, 1=Yes, 999=Unknown	
**Treatment variables**					
	Beta blocker	Beta blockers currently prescribed for heart failure		0=No, 1=Yes, 999=Unknown	
	Calcium channel blocker	Calcium channel blockers currently prescribed for heart failure		0=No, 1=Yes, 999=Unknown	
	Digoxin	Digoxin currently prescribed for heart failure		0=No, 1=Yes, 999=Unknown	
	Diuretics	Diuretics currently prescribed for heart failure		0=No, 1=Yes, 999=Unknown	
**Burden of care**					
	Date of arrival	Date of admittance		DD/MM/YYYY	
	Date of discharge	Date of discharge		DD/MM/YYYY	
	Hospital admissions	Number of hospitalizations in last 12 months due to heart failure		Numerical value or 999=Unknown	
	Hospital appointments	Number of hospital appointments in last 12 months due to heart failure		Numerical value or 999=Unknown	
**Mortality**					
	Date of death	Date patient was declared dead		DD/MM/YYYY or 999=Unknown	

Anonymized data on patients, visits, diagnosis, procedures, drug administration events, laboratory tests and patient measures were collected from the relational database IMASIS-2 where all these fact tables are connected to the patient table via the patient identifier. In addition, visit, diagnosis, and procedures are connected to each other via the visit identifiers, whereas drugs, laboratory, and patient measures are connected to all domains via date matching. Specific queries requesting data from each of these tables yielded the “Temporary datasets” that were subjected to several transformation steps and to a successive left outer join merging process in which patient and visit identifiers were set as the initial left dataset. As a result, data were organized in a “visit-centered” fashion (every row contains all data related to a visit), thus providing the final dataset according to the ICHOM format.

#### Data Quality Dimensions

To evaluate the quality of heart failure patient data collected during routine clinical care, a subset of 5 data quality dimensions was selected: completeness, correctness, consistency, uniqueness, and stability. These dimensions are most commonly used in the data quality literature and were deemed most interesting to assess given the nature of the data.

First, for *uniqueness*, we measured the frequency with which partially duplicated patient records occur. Second, for *consistency*, we assessed data compliance with their expected data type (percentage of fields of a different type than defined), value range (percentage of fields out of the expected range), and basic multivariate rules (percentage of data not fulfilling rules; for example, patient’s arrival date should be before or equal to their date of discharge) [10]. Next, for *completeness,* we measured the proportion of complete fields per variable. Further, for *stability*, we qualitatively evaluated the temporal stability of recorded past medical conditions and usage of different types of medications. To this end, we computed, per month, how many patient visit records mentioned a history of a particular medical condition or usage of a specific medication out of the total number of patient visit records that month. We then visualized trends for each of these data items by plotting the respective relative frequencies over time. Finally, we inferred data correctness from the data, either by combining information across variables or by investigating data from the same patient over time. Specifically, plausibility of height and weight was examined by computing patients’ BMIs. Further, we investigated the temporal order of past medical conditions, assuming that once a hospital visit record indicates that a patient has a history of atrial fibrillation, hypertension, diabetes, or myocardial infarction, the history of this diagnosis or event should be mentioned in all subsequent visit records. Based on this assumption, for assessment purposes, some deviations from this temporal order (ie, “history” followed by “no history”) point to data errors in the extracted dataset.

#### Tools

We conducted the data quality assessment using R, version 3.6.1 [35]. For the temporal stability analyses, we used the EHRtemporalVariability R package [36].

## Results

### Uniqueness

Of a total of 142,345 patient visit records, 1.2% had identical visit identifiers even though values for one or more data items had different inputs (Uniqueness result 1=98.8%). In turn, 2.8% of all patient visit records had at least another record with a different visit identifier registered the same day and identical clinical data (Uniqueness result 2=97.2%). In IMASIS-2, visits and clinical data are connected via date matching. Therefore, for 1 patient attending 2 visits in the same day, both visits are connected to the same data. This amounts to an average score of 98% for uniqueness.

### Consistency

Consistency by type and by multivariate rules both yielded a score of 100%; all values were in the right format, and no errors in relationships between dates were found. As a third consistency check, we examined whether numerical and date values fell within prespecified ranges and whether categorical variables had values that complied with predefined response options. An average score of 91.21% was obtained for consistency by range, resulting from errors in 3 variables. In particular, 85% of values for height and weight were “0.” Since weight and height values of zero do not have a physical meaning, we hypothesized that these data points were missing data values. Indeed, zero entries are not even permitted in the structured data fields of height and weight. Rather, these zero values were introduced during data extraction from the IMASIS-2 database to indicate missingness, since only numeric values are accepted for height and weight according to the ICHOM Heart Failure data dictionary (summarized in Table 1). In addition, a small number of out-of-range data points were identified for height (n=54) and weight (n=20). Further, 16 visit records had arrival dates before January 1, 2006. Across the 3 domains of consistency, this yields an average score of 97.07%.

### Completeness

Assessing completeness of the dataset by column revealed that all included variables were completely documented, except for date of death, which was only recorded in 37.14% of all patient visits. This incompleteness is valid, though, since date of death is only provided when the patient died during the visit. Excluding this valid incompleteness result, an average score of 100% was obtained for completeness.

### Stability

**Figure 2:**
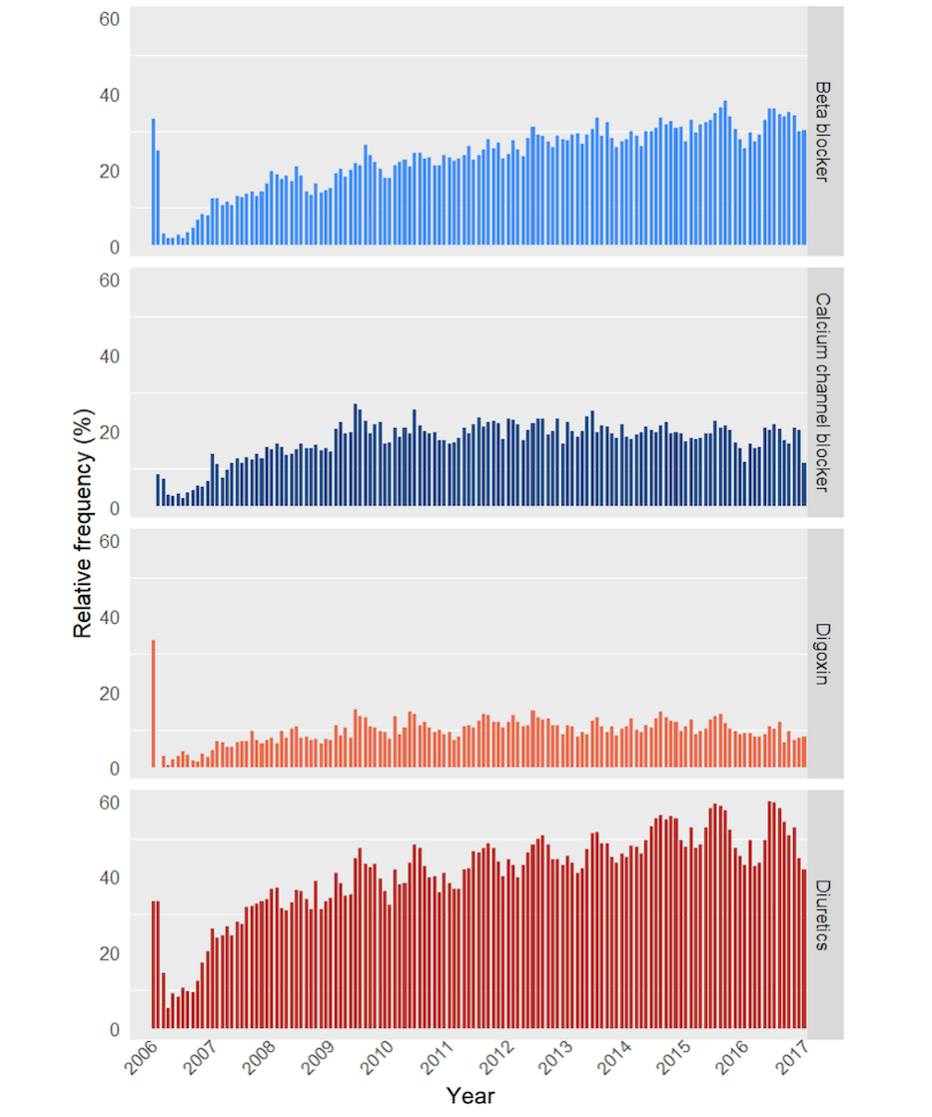
Percentage of patients with a record of specific drug usage per month, relative to the total number of patient admissions within that month, plotted over time.

**Figure 3:**
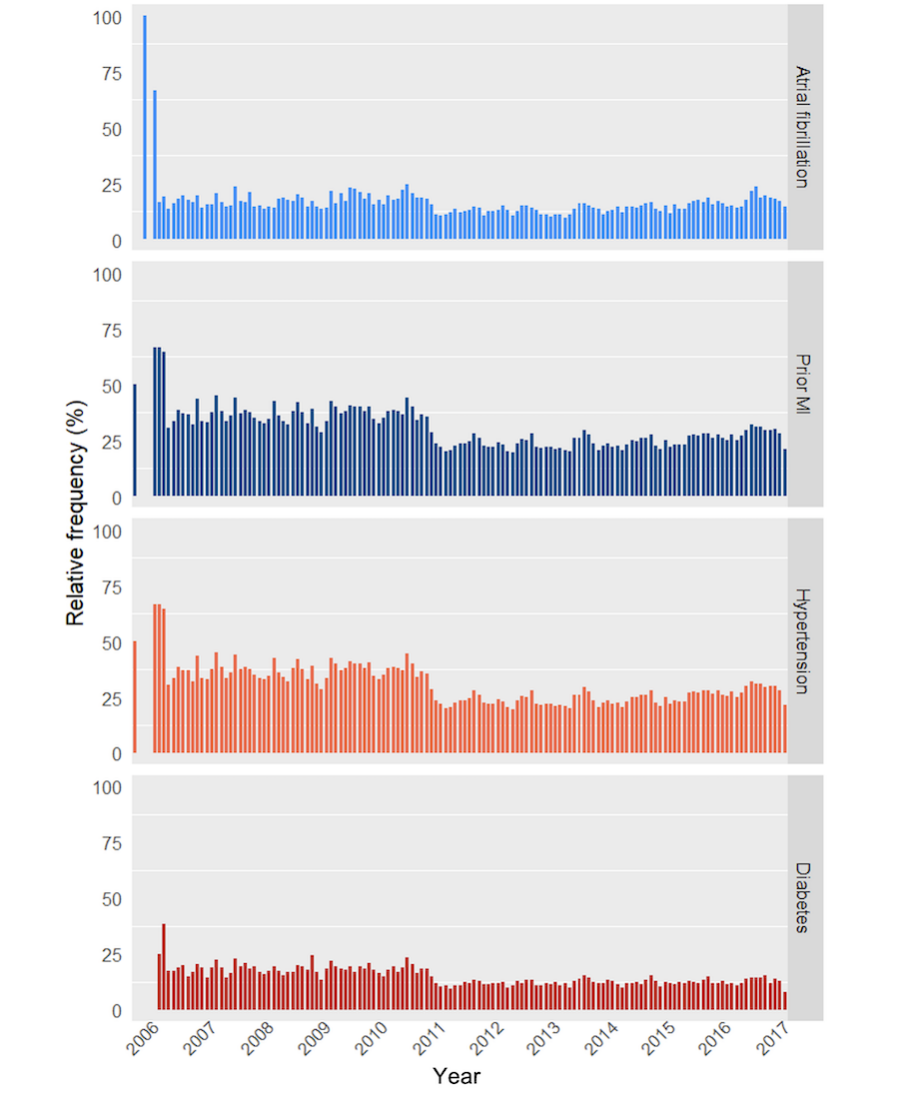
Percentage of patients with a record of a specific past medical condition per month, relative to the total number of patient admissions within that month, plotted over time. MI=myocardial infarction.

Two categories of data items were assessed for temporal variability: medication usage and past medical conditions. As illustrated in Figure 2, the results showed a gradual increase over time in the recorded usage of different types of medication to treat heart failure, especially of beta blockers and diuretics. Further, we found an abrupt change in the documentation pattern of past medical conditions in 2011, with drastically reduced frequencies of reported past medical conditions (Figure 3). Of note, only a small number of patient visit records (<10) was available for each month in the first half of 2016, explaining the absent or divergent results.

### Correctness

**Figure 4:**
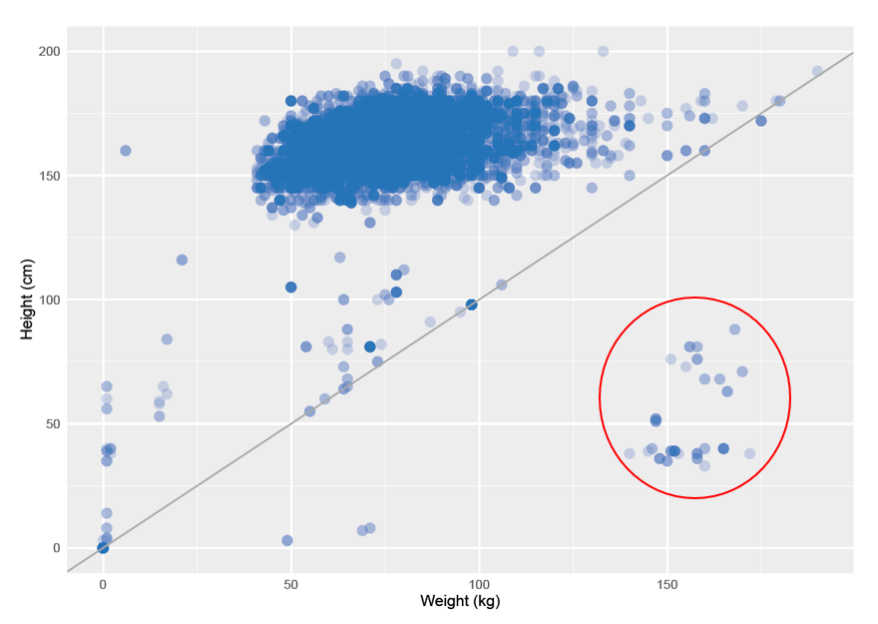
Bivariate distribution of height and weight values, with the red circle highlighting the data points where height and weight values were hypothesized to have been inverted.

After performing basic descriptive analyses, results of which are summarized in Multimedia Appendix 4, 2 sets of variables were subjected to closer inspection. First, correctness of height and weight values was evaluated based on their bivariate distribution, as shown in Figure 4. All data points that fall below the main diagonal, implying that the patient’s weight (in kg) is larger than his or her height (in cm), are very unlikely to be true. A subset of these data errors, highlighted by the red circle, were hypothesized to result from value inversion between height and weight recordings. To formally assess implausible height and weight values, we computed the patients’ BMIs. Results showed that 16 patients had a suspiciously low BMI (<10 kg/m^2^), and 180 patients had an implausibly high BMI (>70 kg/m^2^). Hence, a total of 196 probable errors were identified, corresponding to 0.13% of all patient visit records.

Further, we investigated the temporal order of past medical conditions. Results showed a substantial number of deviations. Specifically, 6.33% of all patient visit records mentioned that the patient did not have a history of atrial fibrillation, while earlier records indicated the patient had previously been diagnosed with atrial fibrillation. Similarly, for history of hypertension, diabetes mellitus, and myocardial infarction, error rates of 12.11%, 6.12%, and 12.11%, respectively, were obtained. These deviations in temporal order were introduced while mapping the IMASIS-2 relational database contents to the ICHOM format, as the latter requires a level of detail that is not explicitly available in the coded data of an EHR. In particular, diagnoses or events already recorded in a previous visit and not mentioned in a subsequent visit are not consistently recorded in EHR systems during routine clinical care, in contrast to data collected for research purposes. It is therefore practically impossible to distinguish true negatives from missing data when extracting data from the EHR. As a result, a substantial proportion of patient history data items that were negative in the dataset actually represent missing data values. Taken together, this amounts to a total score of 93.84% for correctness.

## Discussion

### Data Quality Assessment Results and Suggestions for Improvement

Overall, this pilot assessment revealed high scores on each of the dimensions used to investigate the quality of heart failure patients’ data. Nevertheless, several data quality issues were identified, based on which we propose a set of improvement strategies.

Regarding consistency, results of our data quality assessment showed that a substantial number of negative values in the dataset — indicating the absence of a particular data item — actually represented missing data. Consequently, some variable distributions seem to be biased. For example, according to the data, only a minority of patients currently smoked or had a past medical condition such as hypertension (see Multimedia Appendix 4), which is rather implausible for a population of patients with heart failure. This is an intrinsic issue associated with structured data sources in the framework of EHR databases. That is, when a code is not found in the EHR, it is practically impossible to distinguish whether the code is negative (ie, examination has confirmed the absence of a particular condition) or missing (ie, no examination has taken place, or examination confirmed the presence of a particular condition but is not recorded in a structured format) for a given patient. We are aware that good clinical practice does not mandate the measurement of every data item at each patient visit (eg, disease history), since these items usually are present as additional information in a typical EHR environment. Nevertheless, this differs fundamentally from data collection practices in the context of research activities such as outcomes assessment, for which the ICHOM standard set was originally developed. When performing analytical and research activities, it would therefore be very useful to introduce mechanisms or tools that allow differentiation of data missingness from true negatives and to determine the duration of each condition and disease, regardless of whether they are mentioned in each visit.

Further, the uniqueness analyses revealed some partially duplicated patient visit records. First, duplications in visit identifiers were found, while clinical data showed different inputs. Data management staff at the Hospital del Mar clarified that this happened whenever different height and weight measurements were registered during a single visit. If a slight difference between values is observed, partial row duplicates are generated when merging data in the final dataset. Second, duplicated rows with different visit identifiers have arisen because of the data organization in IMASIS-2, where some clinical data are connected to visit IDs via date matching. As a result, all clinical data collected during different patient visits on the same day are connected to different visit IDs depending on the department or hospital service where these patients visit even on the same day. To reduce future data quality issues of this kind, we suggest a data reorganization including a 2-level visit structure. First, a more general level would describe a period in which one or different visits occur and is connected to clinical data obtained within this period. Second, a more specific level would then describe every distinct visit together with a corresponding diagnosis and procedure information obtained during the particular visit. This 2-level visit organization would contribute to the elimination of partial replicates, thus positively impacting the uniqueness aspect of data quality. This strategy has been previously adopted by the Observational Medical Outcomes Partnership (OMOP) Common Data Model (CDM) standard [37] with the aim of easing mappings from ambiguous visit-connected schemas.

When analyzing and interpreting completeness, it is essential to take into account the type of information that is registered based on the characteristics of the database, for instance, in this case a hospital-based EHR in which information and variables related to death and data for death are only registered when this situation occurs during admission. For instance, the link among different registries and databases such as primary care, hospital, and mortality registries is essential to contribute to the completeness of this type of information.

Temporal stability analyses revealed an abrupt change in the documentation pattern of past medical conditions in 2011, with drastically reduced frequencies of reported past medical conditions. For instance, the introduction of a new automated coding system in the emergency department EHR system accompanied an increase in the number of registries and codifications in this department and therefore in the system. Although we assume this evolution in the recording of past medical conditions had a positive impact on direct patient care, decision support and alert algorithms can be impacted by changes in diagnostic coding practice and should therefore be considered. In addition, these changes will affect the reuse of data for research and quality monitoring such as outcomes tracking. In this sense, quality assessment is an essential tool to detect the effects of changes in EHR systems introduced over time, which would contribute to a better understanding of the updates in the content and structure of these types of databases. Finally, regarding the important point related to the potential impact of changes or upgrades in EHR system and diagnostic coding practices due to common changes in the way diseases are coded or for instance the necessity to included new diseases, we recommend preparing carefully for this type of situation.

In relation to correctness, many data items are often recorded in free text rather than structured data fields, making it difficult to extract this information for research and analysis purposes. We therefore advise to maximally include data items in form format or specific fields or sections in the EHR. In addition, when using form formats, we recommend the use of alarms for avoiding missing values as well as for inputting out-of-range data. Alternatively, natural language processing techniques applied to free-text clinical annotation fields can be used to enrich structured sources.

### Lessons Learned

The process of assessing the quality of outcomes data obtained during routine clinical care is of great value and allows us the opportunity to learn several relevant aspects in the management and evaluation of clinical information in EHR environments. The most relevant lessons learned were (1) the evaluation requires having considerable knowledge of the EHR (data available, how the data were collected, or who collected it) to fully understand its structure and different staff needs; (2) it is critical that the metrics are feasible, valid, and meaningful for a specific EHR system and its quality evaluation and should be understood and used accordingly; (3) once the quality of the data is assessed, it is important to monitor it regularly, and the value of an external data quality assessment by an independent organization should be considered. In addition, high-quality data enhance the validity and reliability of study findings and thinking of using EHR systems for purposes other than health care such as research. Finally, it is interesting to consider that EHR models would need to be expanded and redesigned in content and structure, and a data quality assessment can assist in doing these tasks.

### Limitations and Future Directions

In interpreting the results of this study, some important limitations should be taken into consideration. First, although the selection of a subset of ICHOM outcome variables for the data quality assessment was made in agreement among all the members of the study assessment based on the most likely routinely collected data within their EHR for patients with CHF, it is possible that the use of more variables or other variables could affect the results of the quality assessment. For this reason, whether the data quality results from this pilot assessment are generalizable to the complete ICHOM standard set has yet to be investigated. Similarly, we selected 5 of 9 available data quality dimensions, as these were thought to be most relevant given the nature of the data. It is possible that the use of all 9 dimensions would show a more complete analysis of this type of data and therefore would offer additional recommendations for improvement. Further, data quality assessment was performed on a data extract from the IMASIS-2 dataset after mapping the data items to the ICHOM outcomes format, which might have introduced additional errors. We therefore recommend future studies to examine the data quality of the EHR variables directly, in the hospital’s own response format, or to perform an additional data quality assessment of the mapping procedure.

In sum, future research would benefit from performing more thorough data quality assessments, across multiple hospitals, to truly examine to what extent hospitals today are able to routinely collect the evidence of their success in achieving good health outcomes. The European Federation of Pharmaceutical Industries and Associations (EFPIA) is currently leading such a project together with i~HD. In particular, the goal of this project is to assess the availability and quality of routinely collected patient data to underpin a future scale-up of value-based care models in which ICHOM outcomes indicators serve as the measures of value delivered by health care provider organizations. For this project, data from patients with heart failure are also being examined, now using the complete set of ICHOM outcomes indicators and performing assessments across 10 European hospitals. The promotion of data quality is essential to advance learning health systems, patient empowerment, and clinical research, and the results of this larger project will provide interesting insights on the generalizability of this pilot project’s findings.

## Appendix

Multimedia Appendix 1 Mapping of data quality dimensions.

Multimedia Appendix 2 ICD-9 classification codes used for the evaluation of baseline health status variables.

Multimedia Appendix 3 Anatomical Therapeutic Chemical classification system (ATC/DDD) codes of the World Health Organization used to retrieve patients’ medication usage.

Multimedia Appendix 4 Results of descriptive analyses.

## Abbreviations

AI: artificial intelligence
CDM: Common Data Model
CHF: congestive heart failure
EFPIA: European Federation of Pharmaceutical Industries and Associations
EHR: electronic health record
EMIF: European Medical Information Framework
ICD-9: International Classification of Diseases ninth edition
ICHOM: International Consortium for Health Outcomes Measurement
i~HD: European Institute for Innovation through Health Data
OMOP: Observational Medical Outcomes Partnership

## References

[1] Donabedian A (1966). Evaluating the Quality of Medical Care. The Milbank Memorial Fund Quarterly.

[2] O'Connor DP, Brinker M (2013). Challenges in outcome measurement: clinical research perspective. Clin Orthop Relat Res.

[3] Porter ME, Teisberg EO (2006). Redefining Health Care: Creating Value-Based Competition on Results.

[4] Kelley TA (2015). International Consortium for Health Outcomes Measurement (ICHOM). Trials.

[5] Botsis T, Hartvigsen G, Chen F, Weng C (2010). Secondary Use of EHR: Data Quality Issues and Informatics Opportunities. Summit Transl Bioinform.

[6] Chan KS, Fowles JB, Weiner JP (2010). Review: electronic health records and the reliability and validity of quality measures: a review of the literature. Med Care Res Rev.

[7] Daniel C, Serre P, Orlova N, Bréant S, Paris N, Griffon N (2019). Initializing a hospital-wide data quality program. The AP-HP experience. Comput Methods Programs Biomed.

[8] Doods J, Botteri F, Dugas M, Fritz F, EHR4CR WP7 (2014). A European inventory of common electronic health record data elements for clinical trial feasibility. Trials.

[9] Weir CR, Hurdle JF, Felgar MA, Hoffman JM, Roth B, Nebeker JR (2003). Direct text entry in electronic progress notes. An evaluation of input errors. Methods Inf Med.

[10] Sáez C, Moner D, García-De-León-Chocano R, Muñoz-Soler V, García-De-León-González R, Maldonado JA, Boscá D, Tortajada S, Robles M, García-Gómez JM, Alcaraz M, Serrano P, Bernal JL, Rodríguez J, Bustos G, Esparza M (2017). A Standardized and Data Quality Assessed Maternal-Child Care Integrated Data Repository for Research and Monitoring of Best Practices: A Pilot Project in Spain. Stud Health Technol Inform.

[11] Hirata K, Kang A, Ramirez GV, Kimata C, Yamamoto LG (2019). Pediatric Weight Errors and Resultant Medication Dosing Errors in the Emergency Department. Pediatr Emerg Care.

[12] Selbst SM, Fein JA, Osterhoudt K, Ho W (1999). Medication errors in a pediatric emergency department. Pediatr Emerg Care.

[13] Burns DJ, Arora J, Okunade O, Beltrame JF, Bernardez-Pereira S, Crespo-Leiro MG, Filippatos GS, Hardman S, Hoes AW, Hutchison S, Jessup M, Kinsella T, Knapton M, Lam CS, Masoudi FA, McIntyre H, Mindham R, Morgan L, Otterspoor L, Parker V, Persson HE, Pinnock C, Reid CM, Riley J, Stevenson LW, McDonagh TA (2020). International Consortium for Health Outcomes Measurement (ICHOM): Standardized Patient-Centered Outcomes Measurement Set for Heart Failure Patients. JACC Heart Fail.

[14] Weiskopf NG, Weng C (2013). Methods and dimensions of electronic health record data quality assessment: enabling reuse for clinical research. J Am Med Inform Assoc.

[15] Savarese G, Lund LH (2017). Global Public Health Burden of Heart Failure. Card Fail Rev.

[16] Wang RY, Strong DM (2015). Beyond Accuracy: What Data Quality Means to Data Consumers. Journal of Management Information Systems.

[17] Batini C, Cappiello C, Francalanci C, Maurino A (2009). Methodologies for data quality assessment and improvement. ACM Comput. Surv.

[18] Johnson SG, Speedie S, Simon G, Kumar V, Westra BL (2015). A Data Quality Ontology for the Secondary Use of EHR Data. AMIA Annu Symp Proc.

[19] Kahn MG, Raebel MA, Glanz JM, Riedlinger K, Steiner JF (2012). A pragmatic framework for single-site and multisite data quality assessment in electronic health record-based clinical research. Med Care.

[20] Liaw S, Rahimi A, Ray P, Taggart J, Dennis S, de Lusignan S, Jalaludin B, Yeo A, Talaei-Khoei A (2013). Towards an ontology for data quality in integrated chronic disease management: a realist review of the literature. Int J Med Inform.

[21] Kalra D, Stroetmann V, Sundgren M, Dupont D, Schlünder I, Thienpont G, Coorevits P, De Moor G (2017). The European Institute for Innovation through Health Data. Learn Health Syst.

[22] Zozus M, Hammond W, Green B, Kahn M, Richesson R, Rusincovitch S, Simon G, Smerek M (2014). Assessing Data Quality for Healthcare Systems Data Used in Clinical Research (Version 10).

[23] Davoudi S, Dooling J, Glondys B, Jones T, Kadlec L, Overgaard S, Ruben K, Wendicke A (2015). Data Quality Management Model (2015 Update) - Retired. The American Health Information Management Association.

[24] Sáez C, Martínez-Miranda J, Robles M, García-Gómez JM (2012). Organizing data quality assessment of shifting biomedical data. Stud Health Technol Inform.

[25] Kahn MG, Callahan TJ, Barnard J, Bauck AE, Brown J, Davidson BN, Estiri H, Goerg C, Holve E, Johnson SG, Liaw S, Hamilton-Lopez M, Meeker D, Ong TC, Ryan P, Shang N, Weiskopf NG, Weng C, Zozus MN, Schilling L (2016). A Harmonized Data Quality Assessment Terminology and Framework for the Secondary Use of Electronic Health Record Data. EGEMS (Wash DC).

[26] Bray F, Parkin DM (2009). Evaluation of data quality in the cancer registry: principles and methods. Part I: comparability, validity and timeliness. Eur J Cancer.

[27] Sariyar M, Borg A, Heidinger O, Pommerening K (2013). A practical framework for data management processes and their evaluation in population-based medical registries. Inform Health Soc Care.

[28] Sáez C, Zurriaga O, Pérez-Panadés J, Melchor I, Robles M, García-Gómez JM (2016). Applying probabilistic temporal and multisite data quality control methods to a public health mortality registry in Spain: a systematic approach to quality control of repositories. J Am Med Inform Assoc.

[29] Mitchell E, Loomes KM, Squires RH, Goldberg D (2018). Variability in acceptance of organ offers by pediatric transplant centers and its impact on wait-list mortality. Liver Transpl.

[30] Pagani E, Hirsch JG, Pouwels PJ, Horsfield MA, Perego E, Gass A, Roosendaal SD, Barkhof F, Agosta F, Rovaris M, Caputo D, Giorgio A, Palace J, Marino S, De Stefano N, Ropele S, Fazekas F, Filippi M (2010). Intercenter differences in diffusion tensor MRI acquisition. J Magn Reson Imaging.

[31] Sáez Carlos, García-Gómez Juan M (2018). Kinematics of Big Biomedical Data to characterize temporal variability and seasonality of data repositories: Functional Data Analysis of data temporal evolution over non-parametric statistical manifolds. Int J Med Inform.

[32] Lovestone S, EMIF Consortium (2020). The European medical information framework: A novel ecosystem for sharing healthcare data across Europe. Learn Health Syst.

[33] Roger VL (2013). Epidemiology of Heart Failure. Circ Res.

[34] (2010). Guidelines for ATC classification and DDD assignment, 2011. WHO Collaborating Centre for Drug Statistics Methodology.

[35] (2017). R: A language and environment for statistical computing. R Foundation for Statistical Computing.

[36] Sáez C, Gutiérrez-Sacristán A, Kohane I, García-Gómez JM, Avillach P (2020). EHRtemporalVariability: delineating temporal data-set shifts in electronic health records. Gigascience.

[37] (2021). The Book of OHDSI. Observational Health Data Sciences and Informatics.

