# Supplementary material for: Quality of Hospital Electronic Health Record (EHR) Data Based on the International Consortium for Health Outcomes Measurement (ICHOM) in Heart Failure: Pilot Data Quality Assessment Study
Source: JMIR Med Inform. 2021 Aug 4;9(8):e27842. doi: 10.2196/27842 (PMC8374665; doi:10.2196/27842)
Supplement: Multimedia Appendix 1 [file medinform_v9i8e27842_app1.docx]

**Supplementary Table 1.** Mapping of data quality dimensions.

| Data quality dimension | Weiskopf & Weng, 2013 | Kahn et al., 2012 | Botsis et al., 2010 | Zozus et al., 2014 | Davoudi et al., 2015 | Saez et al.,  2012 | Sariyar et al., 2013 | Bray & Parkin, 2009 | Kahn et al., 2016 |
| --- | --- | --- | --- | --- | --- | --- | --- | --- | --- |
| Completeness | Completeness | Appropriate amount of data | Incompleteness | Completeness | Comprehensive-ness | Completeness | Completeness | Completeness + Validity and accuracy | Completeness |
| Correctness | Correctness | Accuracy | Inaccuracy | Accuracy | Accuracy | Correctness | Accuracy | Validity and accuracy | Atemporal plausibility |
| Consistency | Concordance |  | Inconsistency | Consistency | Consistency | Consistency | Consistency | Validity and accuracy | Conformance & Atemporal plausibility |
| Trustworthiness | Plausibility | Believability |  |  |  | Reliability | Trustworthi-ness |  |  |
| Timeliness | Currency | Timeliness |  |  | Currency/ timeliness |  | Timeliness | Timeliness | Temporal plausibility |
| Uniqueness |  |  |  |  |  | Uniqueness |  |  | Uniqueness plausibility |
| Stability |  |  |  |  |  | Source & temporal stability |  | Comparability | ~(A)temporal plausibility |
| Representative-ness |  |  |  |  | Relevancy | Predictive value | Relevancy |  |  |
| Contextualization |  |  |  |  | Definition | Contextualization |  |  |  |
|  |  |  |  |  |  |  |  |  |  |
